# Engineering affibody domains as anti-idiotypic masks for nivolumab-based prodrugs

**DOI:** 10.1093/protein/gzag010

**Published:** 2026-04-16

**Authors:** Anna Mestre Borras, Hanna Mehari, Stefan Ståhl, John Löfblom

**Affiliations:** Department of Protein Science, School of Engineering Sciences in Chemistry, Biotechnology and Health, KTH Royal Institute of Technology, SE-106 91, Stockholm, Sweden; Department of Protein Science, School of Engineering Sciences in Chemistry, Biotechnology and Health, KTH Royal Institute of Technology, SE-106 91, Stockholm, Sweden; Department of Protein Science, School of Engineering Sciences in Chemistry, Biotechnology and Health, KTH Royal Institute of Technology, SE-106 91, Stockholm, Sweden; Department of Protein Science, School of Engineering Sciences in Chemistry, Biotechnology and Health, KTH Royal Institute of Technology, SE-106 91, Stockholm, Sweden

**Keywords:** nivolumab, prodrug, affibody molecule, PD-1, conditional activation, bacterial display, directed evolution

## Abstract

Antibody prodrugs provide a strategy to reduce systemic toxicity by masking therapeutic antibodies until activation in specific physiological environments. Nivolumab, an anti-PD-1 checkpoint inhibitor used in cancer immunotherapy, can cause immune-related adverse events. As a first step toward a prodrug version of nivolumab, we screened an *Escherichia coli* affibody library using MACS and FACS, identifying affibodies that effectively mask its PD-1-binding regions. Deep sequencing revealed an unexpected enrichment of proline-rich variants, possibly mimicking a PD-1 loop. AlphaFold modeling suggested that these affibodies form interactions with nivolumab despite low alpha-helical content. Biosensor assays confirmed effective masking in a nivolumab prodrug format, with PD-1 binding restored upon proteolytic cleavage. These findings support further exploration of PD-1–mimicking affibodies as masking domains, advancing the development of more selective nivolumab prodrugs with improved safety profiles.

## Introduction

**Figure 1:**
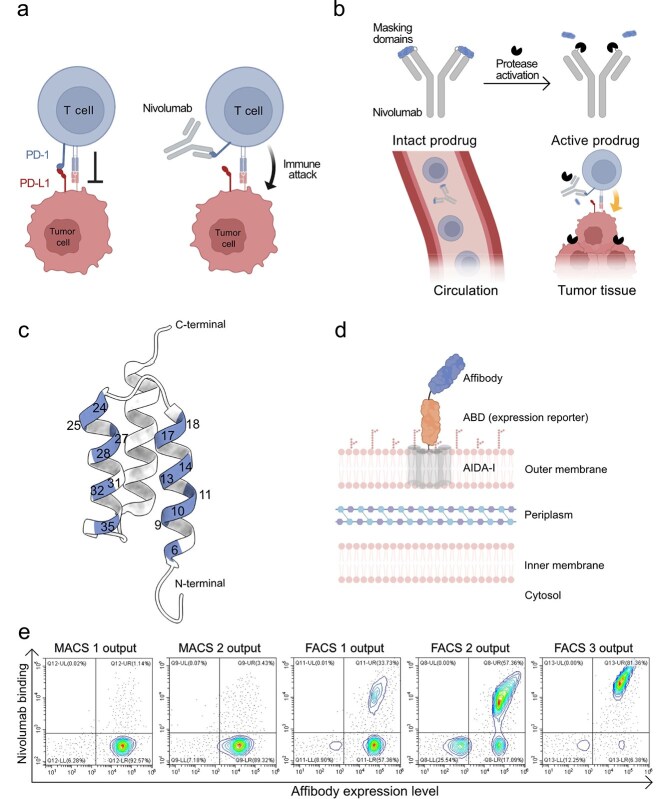
Mechanism of action of nivolumab and affibody selection using *E. coli* display for prodrug masking domain discovery. (a) PD-1 on T cells binds PD-L1 on tumor cells, suppressing immune activation. Nivolumab blocks this interaction, restoring T cell cytotoxicity. (b) Prodrug concept: masking domains are fused to nivolumab to prevent activity in circulation. Protease-mediated cleavage in the tumor microenvironment removes the masking domains, enabling localized immune activation. (c) Structure of the affibody scaffold (PDB: 2KZJ), with randomized positions in the library highlighted. (d) Schematic representation of affibody display on *E. coli*. ABD: albumin-binding domain. Affibody–ABD fusion proteins are anchored to the outer membrane *via* the AIDA-I autotransporter. (e) Flow cytometry contour plots showing enrichment of nivolumab-binding affibody variants during selection. The x-axis indicates affibody surface expression level (ABD detection *via* fluorescent HSA); the y-axis shows nivolumab binding. MACS and FACS outputs from sequential selection rounds are shown.

Antibody prodrugs represent an innovative strategy to enhance the safety and selectivity of therapeutic antibodies, particularly in cancer immunotherapy. By masking the active binding regions with a removable molecular component, prodrugs can limit systemic exposure to the antibody's activity, thereby reducing toxicity (Lucchi et al., 2021). The therapeutic potential of such prodrugs lies in their selective activation in specific tissues, such as the tumor microenvironment. Activation can occur through various mechanisms, including protease-mediated cleavage of the masking moiety, which restores the antibody’s function at the target site (Poreba, 2020; Kavanaugh, 2020). The monoclonal antibody nivolumab has demonstrated remarkable clinical efficacy in treating a range of cancers, including non-small cell lung cancer and melanoma. Its therapeutic effect is achieved by blocking programmed death-1 protein (PD-1)–mediated inhibitory signaling, a pathway that tumors exploit to evade immune detection (Guo et al., 2017; Ferris et al., 2016). By inhibiting this checkpoint, nivolumab restores the anti-tumor activity of T cells and other immune effector cells, thereby enhancing the immune system’s ability to recognize and eliminate cancer cells (Fig. 1a) (Guo et al., 2017; Ferris et al., 2016). However, as with other immune checkpoint inhibitors, widespread systemic activation of immune responses can lead to significant immune-related adverse events which may result in treatment discontinuation (Guo et al., 2017; Shimabukuro-Vornhagen et al., 2018). Engineering nivolumab into a prodrug format, in which its binding regions are masked until activation by tumor-associated proteases, offers a promising approach to reduce systemic autoimmune adverse effects while maintaining therapeutic efficacy (Fig. 1b).

Affibody molecules are engineered proteins that have emerged as attractive alternatives to monoclonal antibodies due to their high specificity, small size, and stable structure (Fig. 1c) (Löfblom et al., 2010; Ståhl et al., 2017). In this study, we aimed to develop a prodrug version of nivolumab by generating affibody molecules that could effectively mask its complementarity-determining regions (CDRs), preventing interaction with PD-1 until unmasking occurs *via* proteolytic cleavage. Using an *Escherichia coli* display system (Fig. 1d) (Parks et al., 2024), we screened an affibody library containing approximately 10^11^ variants to discover binders to nivolumab. Through a combination of magnetic-activated cell sorting (MACS) and fluorescence-activated cell sorting (FACS), we enriched the library for affibodies that specifically bind to nivolumab’s variable region while depleting variants interacting with its constant regions. Unexpectedly, deep sequencing of the selected affibodies revealed the enrichment of proline-rich variants, despite proline residues not being part of the original library design. This seemingly serendipitous finding led us to investigate the structural and functional implications of these proline insertions. Notably, the proline-rich affibodies appeared to mimic a proline motif naturally found in PD-1 (Tan et al., 2017), potentially aiding in their competition with PD-1 for binding to nivolumab. The combined results from flow cytometry, AlphaFold3 structural modeling (Abramson et al., 2024), circular dichroism spectroscopy and bio-layer interferometry suggested that these proline-rich affibodies adopt unique structural features that allow them to effectively block nivolumab’s interaction with PD-1. Moreover, these affibodies showed promise as masking agents in a prototype nivolumab prodrug. The prodrug remained blocked from binding PD-1 until protease treatment, which subsequently activated the nivolumab function. These results highlight the potential of affibodies as versatile masking domains in antibody prodrug constructs, presenting a promising strategy to improve both the safety and efficacy of nivolumab therapies.

## Methods

### Proteins and protein labelling

Nivolumab (Opdivo®, Apotea, Sweden) was biotinylated using the Biotin-XX Microscale Protein Labeling Kit (Invitrogen, USA) following manufacturer’s instructions. Human serum albumin (HSA) (Albumina Kabi 20%, Kabivitrum, Sweden) was labeled with Alexa Fluor 647 (Alexa Fluor™ 647 NHS Ester Succinimidyl Ester, Invitrogen, USA). Protein concentrations were measured by absorbance at 280 nm using a spectrophotometer (Thermo Fisher, USA). Recombinant Human PD-1 Protein (ECD, His Tag) was purchased from Sino Biological (China), and recombinant tobacco etch virus (TEV) protease was produced and purified in-house following standard methods.

### Bacterial culture and library expression

*E. coli* BL21 (DE3) cells transformed with pPALU (Parks et al., 2024) plasmids encoding for an affibody library (~10^11^ variants) were grown overnight (O/N) in Luria-Bertani (LB) medium supplemented with carbenicillin (100 μg/ml). The culture was diluted in fresh LB medium to OD_600_ = 0.1 and grown at 37°C, 150 rpm until OD_600_ = 0.7. Cells were induced with L-arabinose (0.6% final concentration; L (+)-Arabinose, Thermo Scientific Chemicals, USA) and incubated at 37°C, 150 rpm, and the cells were ready to use after 16 h of induction.

### Library selections against nivolumab using magnetic-activated cell sorting (MACS)

Protein A-coated Dynabeads (Invitrogen, USA) were washed twice with PBS-P (phosphate-buffered saline with 0.1% Pluronic® F108 NF Surfactant, pH 7.4, BASF Corporation, USA) and labelled with nivolumab or a non-related mix of human polyclonal IgG (Octagam; Octapharma, Sweden) for 1 h at room temperature (RT) with gentle rotation. *E. coli* cells covering the library 2x were washed and resuspended in PBS-P. Cells were first incubated with 100 μl of uncoated beads in a final volume of 20 ml for 30 min at RT with 15 rpm rotation (first negative selection). Cells in the supernatant were collected and then incubated with 100 μl of IgG-mix beads in a final volume of 20 ml for 1 h at RT (second negative selection). The cells in the supernatant were collected and incubated with nivolumab-coated beads for 1 h at RT (positive selection). The cell vials were placed on a magnetic rack for 10 min to capture the beads, the supernatant was discarded, and the beads were washed with 20 ml PBS-P. Bead capture and washing was repeated three times. Lastly, the bead-bound cells were resuspended in 50 ml of LB medium supplemented with carbenicillin (100 μg/ml). The cultures were incubated O/N at 37°C and 150 rpm. Serial dilutions of samples taken after the magnetic sorting were spread on agar plates to calculate the library size after each round. The MACS selection was repeated, adjusting the volumes to the number of cells, aiming to cover the library 10 times. To evaluate enrichment, 10 μl of induced O/N-grown libraries were washed with 200 μl of ice-cold PBS-P and incubated with 50 nM biotinylated nivolumab for 1 h at RT, 150 rpm. Cells were washed twice with ice-cold PBS-P and labeled with 225 nM Alexa Fluor 647-HSA and 33.3 nM streptavidin R-phycoerythrin conjugate (SAPE; Thermo Fisher Scientific, USA). After another wash with ice-cold PBS-P, cells were resuspended in 200 μl of PBS-P and analyzed using a CytoFLEX S flow cytometer (Beckman Coulter, USA).

### Library selections against nivolumab using fluorescence-activated cell sorting (FACS)

Induced *E. coli* cultures were grown O/N and diluted in PBS-P to achieve 10x library coverage. Cells were washed twice with 800 μl ice-cold PBS-P and incubated with biotinylated nivolumab for 1 h at RT with gentle rotation (15 rpm). Initially, 150 nM of biotinylated antibody were used for the first sorting, followed by 50 nM for the subsequent sortings. Antibody concentration was reduced across selection rounds to increase stringency. Cells were thereafter washed, labeled with 225 nM Alexa Fluor 647-HSA and 33.3 nM SAPE for 30 min at 4°C, and sorted using a CytoFLEX SRT (Beckman Coulter, USA). After sorting, cells were grown O/N in LB medium with carbenicillin (100 μg/ml) at 37°C, 150 rpm, and aliquots were stored in 20% glycerol at −80°C. The library outputs were analyzed by flow cytometry, as described above.

### Deep sequencing of libraries

Plasmids were extracted from O/N cultures of the original and sorted libraries using the Qiagen Miniprep Kit (Qiagen, USA). Amplification of the affibody-coding regions was performed by polymerase chain reaction (PCR) using Phusion High-Fidelity polymerase (Thermo Fisher, USA) with primers containing TruSeq indexes and adapters (Illumina, USA). PCR products were purified with a QIAquick PCR Purification Kit (Qiagen, Germany), and DNA concentration was measured using a Qubit fluorometer (Thermo Fisher, USA). The diversity of the sorted libraries was analyzed by deep sequencing on an Illumina MiSeq system (Illumina, USA) at the National Genomics Infrastructure (NGI, Sweden), following the manufacturer’s protocol. Between 1.8–0.3 million sequences were obtained from each individual sample. The libraries were annotated to match the affibody scaffold and clustered in sequence groups with 100% identity on the affibody variable regions. The amino acid frequency in each affibody variable position was calculated for the 10 largest clusters throughout the selection process. The data was processed using PipeBio (Benchling, USA), Python (Python version 3.9.12, www.python.org, US), Microsoft Excel (Version 16.86; Microsoft Corporation, USA) and GraphPad Prism 10 (GraphPad, USA). Sequencing data was analyzed using PipeBio (version 1.0.5, Benchling, USA) and processed using Python (version 3.9.12, www.python.org), Microsoft Excel (Version 16.86; Microsoft Corporation, USA) and GraphPad Prism 10 (GraphPad, USA).

### Single candidate analysis by flow cytometry

Individual affibody candidates were randomly picked from agar plates and grown O/N in LB medium at 37°C, 150 rpm. Cells were induced with 0.6% L-arabinose as described for the library. After washing with ice-cold PBS-P, 10 μl of each culture were incubated with 25 nM biotinylated nivolumab or 25 nM biotinylated nivolumab pre-incubated with 100 nM PD-1 for 1 h at RT. Cells were washed twice with ice-cold PBS-P and labeled with 225 nM HSA-Alexa Fluor 647 and 33.3 nM SAPE for 30 min at 4°C. Flow cytometry was performed using a CytoFLEX S instrument.

### Recombinant production of affibodies

The DNA sequences of selected affibodies were identified by Sanger sequencing (Microsynth, Switzerland) and cloned into the pET45+ bacterial expression vector (Addgene, USA) using In-Fusion cloning (Takara, Japan). Transformed *E. coli* BL21 (DE3) cells were grown O/N in TSB-Y (Tryptic Soy Broth with yeast extract) medium supplemented with 100 μg/ml carbenicillin at 37°C. The cultures were diluted to OD_600_ = 0.1 and grown at 37°C, 150 rpm until OD_600_ = 0.7. Protein expression was induced with 1 mM isopropyl β-D-1-thiogalactopyranoside (IPTG) and incubated O/N at 25°C, 150 rpm. Cells were harvested by centrifugation at 5000 × g for 10 min, lysed by sonication (1.5 min, 1 sec ON/1 sec OFF), and clarified by centrifugation (10 000 × g, 4°C, 20 min). Proteins were purified using immobilized metal affinity chromatography (IMAC) on cobalt resin (TALON®; Cytiva, Sweden), washed with 50 mM Na_2_HPO_4_, 500 mM NaCl, 15 mM imidazole (pH 7.2), and eluted with 300 mM imidazole. The buffer was exchanged to PBS (pH 7.4) using PD-10 desalting columns (GE Healthcare, Sweden) and the proteins were analyzed by mass spectrometry (4800 MALDI TOF/TOF; Applied Biosystems, USA) and SDS-PAGE (NuPAGE; Invitrogen, USA). Circular dichroism spectroscopy was conducted using a Chirascan spectropolarimeter (Applied Photophysics, UK) to evaluate the protein conformation and thermostability.

### SPR analysis of affibody candidates

The kinetics of the recombinantly produced affibody candidates toward nivolumab were investigated by surface plasmon resonance (SPR) using a Biacore 8 K instrument (Cytiva, Sweden). Nivolumab was immobilized by amine coupling on different channels of a CM5 sensor chip (Cytiva, Sweden) according to the manufacturer’s recommendations. The binding of the affibody candidates was analyzed by multi-cycle kinetics, injecting a serial dilution of each candidate (5000 nM to 21 nM in 1:3 dilutions) over immobilized nivolumab. The experiment was performed at 25°C with a flow rate of 30 μl/min. The chip surface was regenerated by injecting regeneration buffer (10 mM NaOH, and 1 M NaCl) at 30 μl/min for 30 s. HBS-EP+ (0.01 M HEPES, 0.15 M NaCl, 0.003 M EDTA, 0.05% Tween-20, pH 7.4) was used as a running buffer, and the sensorgram curves were fitted using a Langmuir 1:1 model.

### *In silico* protein structure predictions

Protein structure predictions were generated using AlphaFold3 (Abramson et al., 2024; Meng et al., 2023) and visualized using ChimeraX software (Meng et al., 2023). The PD-1-nivolumab complex was retrieved from the Protein Data Bank (PDB ID: 5WT9) (Tan et al., 2017; Berman et al., 2000).

### Cloning and production of Nivolumab prodrugs

The heavy and light chain DNA sequences of nivolumab were cloned into a pcDNA3-derived vector (Invitrogen, USA). To clone the prodrug constructs, the genes for the masking affibodies were fused to the heavy chain genes of nivolumab by an encoded flexible G_4_S linker that includes a sequence cleavable by TEV protease (ENLYFQG). Plasmids were transfected into ExpiCHO cells (ExpiCHO-S™; Gibco, USA) using ExpiFectamine (Gibco™ ExpiFectamine™ CHO Transfection Kit) following manufacturer’s instructions. After 12 days of production, cells were harvested, and supernatants were purified by affinity chromatography using a Protein A column (HiTrap MabSelect PrismA; Cytiva, Sweden). Buffer exchange to PBS was performed using PD-10 desalting columns, and protein purity was confirmed by SDS-PAGE. In order to cleave off the masking domains, the prodrugs were treated with 1 unit of TEV protease for every 3 μg of prodrug in PBS for 12 h at 30°C. Thermal stability of antibody constructs was assessed using nano differential scanning fluorimetry (nanoDSF) on a Prometheus NT.48 instrument (NanoTemper Technologies). Samples were diluted to approximately 0.4 mg/ml in PBS and loaded into standard capillaries. Intrinsic tryptophan fluorescence (350/330 nm ratio) was monitored during a temperature ramp to determine melting temperatures.

### Size exclusion chromatography analysis of prodrugs

The potential aggregation behaviour of the antibody prodrug was evaluated by size exclusion chromatography (SEC) using a Bio-Rad NGC Chromatography System (Bio-Rad, USA), equipped with a Superdex 200 Increase 5/150 GL column (Cytiva, Sweden). Sample volumes of 25 µl were injected onto the column at a concentration between 0.2–0.08 g/l, at a flow rate of 0.45 ml/min. The elution profile of the prodrugs was compared with the profile of a calibrant (thyroglobulin 669 kDa, aldolase 158 kDa, conalbumin 75 kDa, ovalbumin 44 kDa, carbonic anhydrase 29 kDa, and ribonuclease A 13.7 kDa). The column was equilibrated with PBS, and samples were analyzed at a wavelength of 280 nm.

### SPR analysis of nivolumab prodrugs

The masking ability of the intact prodrugs compared to nivolumab was investigated by SPR using a Biacore 8 K instrument. Nivolumab, as well as each of the prodrug constructs were immobilized by amine coupling on different channels of a CM5 sensor chip according to the manufacturer’s recommendations. The masking abilities were analyzed by single cycle kinetics, injecting a serial dilution of PD-1 (100 nM to 3.125 nM in 1:2 dilutions) over immobilized nivolumab and prodrugs. The experiment was performed at 25°C with a flow rate of 30 μl/min. The chip surface was regenerated by injecting regeneration buffer at 30 μl/min for 30 s. HBS-EP+ was used as a running buffer, and the sensorgram curves were fitted using a Langmuir 1:1 model.

### Biosensor evaluation of prodrugs

The prodrug activation and its ability to regain target binding were evaluated using Biolayer Interferometry (OCTET, Sartorius, Germany) with protein A tips (Octet ProA biosensors, Sartorius, Germany). A 10 μg/ml solution of prodrug antibodies was used for capture on the tips, and a 100 nM PD-1 solution was used to assess antibody binding. Nivolumab was used as a control.

### Other tools

Schematic figures were created using BioRender (BioRender.com) and GraphPad Prism 10 (GraphPad, USA) was used to plot and analyze data. AlphaFold 3 was used to predict the affibody protein structures and the interactions with nivolumab Fab (Abramson et al., 2024). UCSF ChimeraX program was used to visualize the protein structure predictions (Meng et al., 2023).

## Results

### Selection of affibody molecules against nivolumab

To enable the development of masking domains for constructing conditionally activated prodrugs based on nivolumab (Fig. 1a and b), we performed selections to obtain affibody molecules with specific binding to its complementarity-determining regions (CDRs). For this purpose, we employed a previously developed *E. coli*-displayed affibody library comprising approximately 10^11^ sequence variants (Fig. 1c–d) (Parks et al., 2024). The affibody library is randomized in 15 surface-exposed positions on helix 1 and helix 2, including all amino acids except proline, glycine, methionine, asparagine and cysteine (Fig. 1c). In this method, each *E. coli* cell displays multiple copies of an affibody variant fused to an albumin-binding domain (ABD) (Andersson et al., 2019; Nilvebrant & Hober, 2013), which serves as a reporter tag to monitor the surface expression level during flow cytometry and cell sorting (Fig. 1e). The library was first enriched for binders using magnetic-activated cell sorting (MACS). To reduce non-specific binders and affibodies recognizing constant regions of antibodies, negative selections were carried out using uncoated beads and beads coated with an unrelated IgG mix. For positive selections, biotinylated nivolumab was immobilized on magnetic beads and mixed with the affibody-displaying *E. coli* library. Flow cytometry analysis showed that successive rounds of MACS effectively reduced the library size and increased the number of binders (Fig. 1e). Following MACS, three additional selection rounds were performed using fluorescence-activated cell sorting (FACS). The *E. coli* library was incubated with biotinylated nivolumab and labeled with two fluorescent reagents, one to detect affibody surface expression *via* the ABD fusion, and the other to detect binding to nivolumab. To increase selection stringency, the concentration of nivolumab was progressively decreased. Flow cytometry confirmed enrichment of nivolumab-binding clones throughout the selection process (Fig. 1e).

### Deep sequencing of sorted libraries

**Figure 2:**
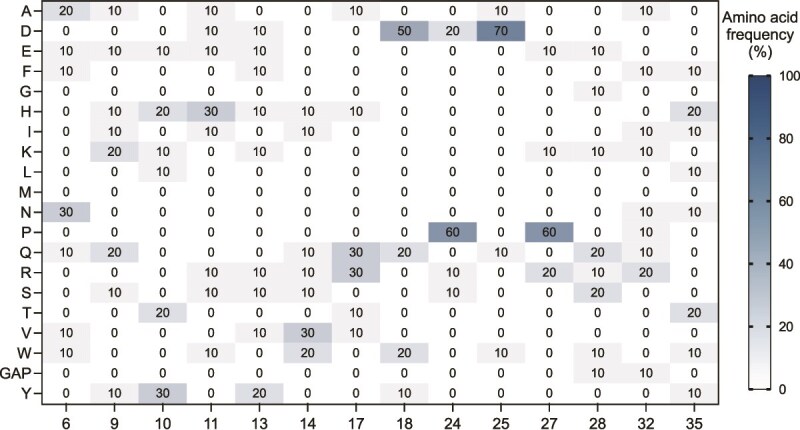
Sequence enrichment analysis of affibody variants after FACS selection. Amino acid frequencies at randomized positions in the 10 most abundant affibody clusters selected against nivolumab. Data are shown as percentages per position, with color intensity reflecting amino acid prevalence. Notably, strong enrichment of proline residues was observed at positions 24, 27, and 32, despite not being part of the original library design.

The sorted libraries were analyzed by deep sequencing with a focus on enriched sequence clusters. The data revealed the emergence of several unintended proline residues at randomized positions in multiple high-ranking hits (Fig. 2). Among the 10 most abundant clusters, 6 contained a proline at randomized position 24, and a similar proportion showed a proline at position 27. Prolines were also observed at position 32, although less frequently than at positions 24 and 27. These findings indicate a consistent enrichment of proline residues across several dominant sequence clusters, despite prolines not even being included in the original library design. In addition to prolines, sequence deletions (gaps) were detected in functionally important regions. Together, these alterations suggest that the presence of prolines, structural flexibility, or a combination of both may confer a selective advantage for these affibodies in binding to nivolumab.

### Single candidates analyzed by flow cytometry

**Figure 3:**
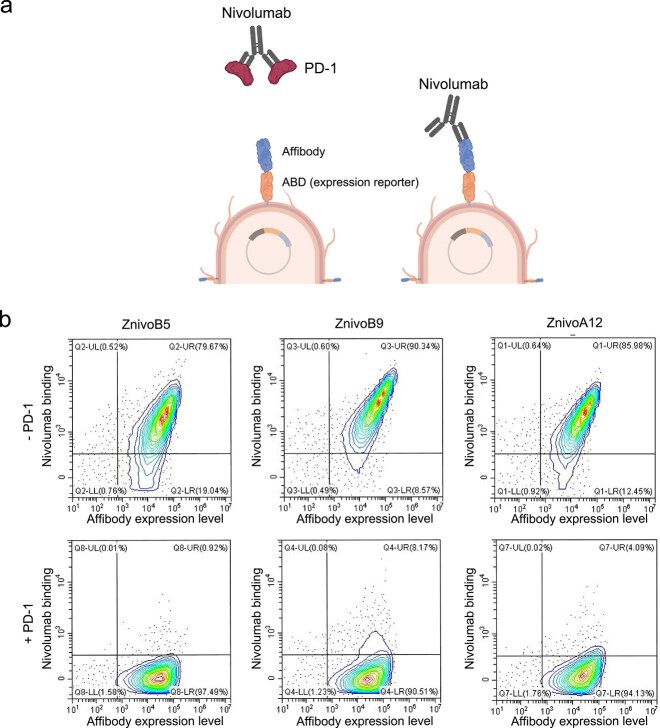
Screening of affibody candidates for masking activity using the *E. coli* display system. (a) Schematic illustration of the *E. coli* display assay used to assess the ability of individual affibody candidates to bind nivolumab in the absence or presence of its antigen, PD-1. Candidate masking affibodies are expected to bind free nivolumab but not nivolumab pre-incubated with PD-1, indicating competition for overlapping epitopes. (b) Flow cytometry contour plots showing binding of affibody candidates ZnivoB5, ZnivoB9, and ZnivoA12 to nivolumab alone (–PD-1) or pre-incubated with PD-1 (+PD-1). The x-axis represents affibody surface expression level (ABD detection), and the y-axis represents nivolumab binding. Reduced binding in the presence of PD-1 suggests epitope overlap between the affibody and PD-1 on nivolumab. Schematics created with BioRender.com.

Nine affibody candidates, representing both unique and recurrent variants, were selected for further characterization by flow cytometry. These candidates contained either none, two, or three prolines at randomized positions. To assess their masking capacity, *E. coli* cells expressing each affibody were incubated either with nivolumab alone or with nivolumab pre-incubated with PD-1, enabling high-throughput evaluation of competitive binding (Fig. 3a). The binding profiles of all screened candidates are summarized in Fig. S1. Representative flow cytometry data for three selected candidates are shown in Fig. 3b: ZnivoB5 (two prolines), ZnivoB9 (three prolines), and ZnivoA12 (a variant with a more conventional sequence lacking prolines at key positions). All three candidates demonstrated a negative shift in fluorescence signal upon addition of PD-1, indicating effective masking of nivolumab. The corresponding amino acid sequences for these variants are provided in Fig. S2.

### Characterization of secondary structure content and refolding

**Figure 4:**
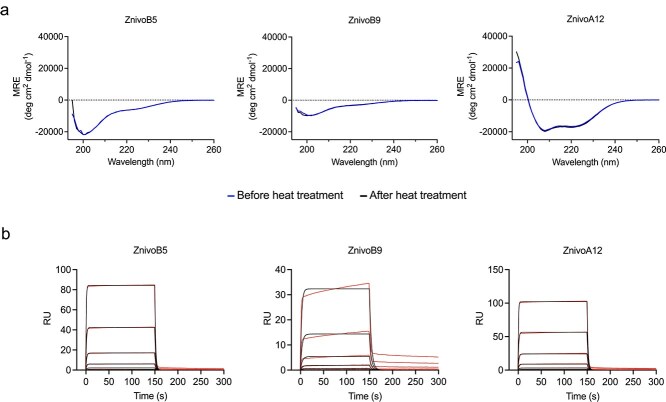
Circular dichroism (CD) analysis and SPR binding analysis of affibody candidates. (a) CD spectra of affibody variants ZnivoB5, ZnivoB9, and ZnivoA12 recorded before and after heat treatment. The x-axis represents wavelength (nm), and the y-axis shows ellipticity (mdeg). Blue traces correspond to spectra before heating, and black traces correspond to spectra recorded after heat treatment. (b) SPR sensorgram showing the binding kinetics between the affibody candidates toward immobilized nivolumab. The x-axis represents time (s), and the y-axis shows the response unit (RU). Sensorgram is shown in red, and the fitted curve is shown in black. Affibody concentrations ranged from 5000 nM to 21 nM.

The three masking domains were successfully expressed and purified as soluble proteins (Fig. S3). Their secondary structure content and refolding properties were analyzed using circular dichroism spectroscopy (CD) (Fig. 4). The CD spectra of the two affibodies containing proline motifs (ZnivoB5 and ZnivoB9) revealed reduced alpha-helical content, suggesting that the proline residues disrupt the formation of one or more alpha helices. In contrast, the ZnivoA12 variant, which lacks proline residues at randomized positions, exhibited a CD spectrum characteristic of the canonical three-helix bundle typically observed in affibody scaffolds. Furthermore, the spectrum of A12 showed no detectable changes before and after heat treatment, indicating efficient refolding following thermal denaturation.

### SPR analysis of affibody candidates

Surface plasmon resonance (SPR) was used to evaluate the binding interaction between the affibody candidates (ZnivoB5, ZnivoB9, and ZnivoA12) and the immobilized ligand nivolumab. Six concentrations of the different affibody candidates were injected over the sensor chip and the results confirm binding of the proteins to the target (Fig. 4b) with similar binding profiles, defined by rapid association and dissociation rates. The data reveal equilibrium dissociation constants (K_D_) in the micromolar range, spanning from around 3.3 to 7.9 μM (Table 1). ZnivoA12, the variant which lacks proline residues at randomized positions, demonstrated the highest binding affinity toward nivolumab.

**Table 1:** Kinetic parameters of affibody masking candidates binding to nivolumab.

Interaction	Binding model	k_a_ (M^−1^ s^−1^)	k_d_ (s^−1^)	K_D_ (μM)	R_max_ (RU)	Chi^2^
ZnivoB5 - nivolumab	1:1 Langmuir	1.16 × 10^5^	5.52 × 10^−1^	4.8 ± 0.10	163	0.46
ZnivoB9 - nivolumab	1:1 Langmuir	3.20 × 10^4^	2.50 × 10^−1^	7.9 ± 0.4	85	3.32
ZnivoA12 - nivolumab	1:1 Langmuir	1.49 × 10^5^	4.95 × 10^−1^	3.3 ± 0.06	169	1.42

Kinetic rate constants and equilibrium dissociation constants (K_D_) were determined by SPR (n = 3, mean ± SD).

### Model of the complex structures

**Figure 5:**
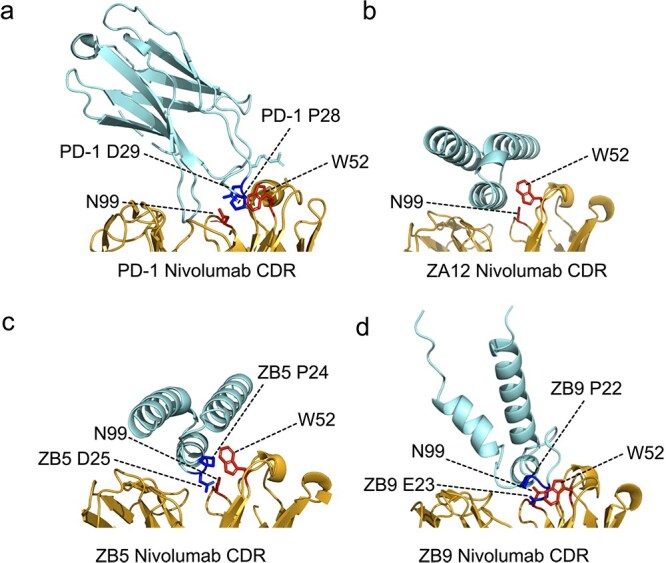
AlphaFold3-predicted interactions between affibody candidates and nivolumab Fab (fragment antigen-binding region). (a) Crystal structure of the PD-1–nivolumab complex (PDB: 5WT9), highlighting the PD-1 N-loop motif residues P28 and D29 (blue) in contact with nivolumab CDR residues W52 and N99 (red). PD-1 is shown as a cyan cartoon and nivolumab Fab as a gold cartoon. (b–d) AlphaFold3-predicted models of affibody variants in complex with nivolumab Fab. Affibody molecules are shown as a cyan cartoon and nivolumab Fab as a gold cartoon. (b) ZnivoA12 (ipTM = 0.76, pTM = 0.82), (c) ZnivoB5 (ipTM = 0.79, pTM = 0.84), and (d) ZnivoB9 (ipTM = 0.79, pTM = 0.84). The affibody motif residues P24 and D25, as well as P22 and E23, respectively, are shown in blue in proximity to nivolumab CDR residues W52 and N99 in red.

Structural models of the interactions between the masking domains (ZnivoB5, ZnivoB9, and ZnivoA12) and the variable region of nivolumab were generated using AlphaFold3 (Abramson et al., 2024) (Fig. 5). All three models yielded predicted TM (pTM) scores above 0.8 (ZnivoA12: 0.82; ZnivoB5: 0.84; ZnivoB9: 0.84), indicating high confidence in the overall predicted structures. The interface predicted TM (ipTM) scores were somewhat lower (0.76–0.79), which may reflect the presence of flexible or partially disordered regions at the binding interface. To explore the potential relationship between affibody binding and PD-1 recognition, the predicted affibody–nivolumab complexes were compared with the experimentally resolved PD-1–nivolumab complex (PDB: 5WT9) (Fig. 5a) (Tan et al., 2017). In the crystal structure, PD-1 engages the nivolumab paratope in part through an N-loop containing residues P28 and D29, which contact a defined set of nivolumab residues (Supplementary Table 1) (Tan et al., 2017). Several of these residues, including W52 and N99, are located within the CDR region and represent key interaction hotspots (Tan et al., 2017). Sequence analysis of the selected affibody variants revealed enrichment of proline residues within the diversified binding surface. Notably, ZnivoB5 contains a P24-D25 motif that resembles the PD-1 P28-D29 motif, whereas ZnivoB9 contains a related P22-E23 motif. The AlphaFold3 models suggest that these motifs are positioned in proximity to overlapping sets of nivolumab residues, including W52 and N99 (Fig. 5c–d; Supplementary Table 1), consistent with engagement of a similar region of the paratope. Despite the presence of helix-disrupting prolines, ZnivoB5 and ZnivoB9 are predicted to form stable interactions with the nivolumab variable region. In contrast, ZnivoA12 appears to retain a more canonical three-helix affibody fold while positioning its binding surface toward the CDR region of nivolumab (Fig. 5b), also contacting residues such as W52 and N99. These observations suggest that different structural solutions may converge on a similar functional binding region. Taken together, the structural models are consistent with the affibody variants engaging the antigen-binding region of nivolumab in a manner compatible with competition with PD-1. Because the models are based on computational predictions, they should be interpreted as structural hypotheses that are consistent with the experimental binding data rather than definitive structural evidence of the interaction mechanism.

### Production and evaluation of nivolumab prodrugs

**Figure 6:**
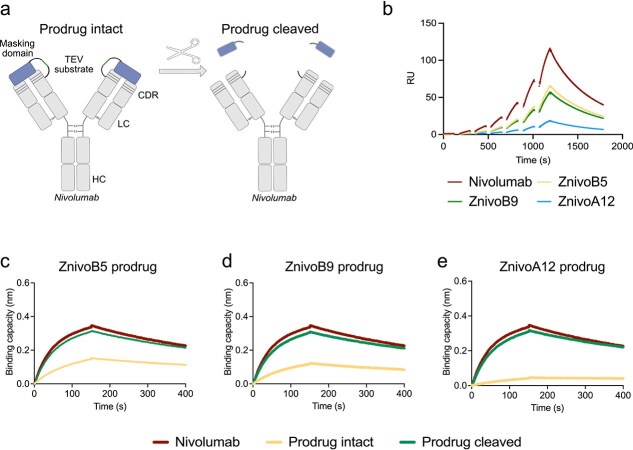
SPR and biolayer interferometry analysis of PD-1 binding by intact and cleaved nivolumab prodrugs. (a) Schematic of the prodrug design. Masking domains (blue) are fused to nivolumab *via* a protease-cleavable linker. Upon protease treatment, the masking domain is removed, restoring access to the antigen-binding site. HC: heavy chain; LC: light chain; CDR: complementarity-determining region. (b) SPR sensorgram showing the binding kinetics between PD-1 and immobilized nivolumab and intact prodrugs. The x-axis represents time (s), and the y-axis shows the response unit (RU). Data points in the dissociation phase have been removed due to air spikes in the instrument. PD-1 concentrations ranged from 100 nM to 3.125 nM. (c–e) BLI sensorgrams showing the binding of PD-1 to intact and cleaved prodrugs based on ZnivoB5 (c), ZnivoB9 (d), and ZnivoA12 (e). Prodrugs were tested in their intact (yellow lines) and cleaved (green lines) forms. Parental nivolumab (red lines) was included as a control. Cleavage of the masking domain results in restored PD-1 binding, confirming functionality of the prodrug design.

Nivolumab-based prodrugs were created by fusing the masking affibodies (ZnivoB5, ZnivoB9, and ZnivoA12) to the N-terminus of nivolumab's heavy chain using a flexible (G4S) linker that incorporated a protease substrate sequence (Figs 6a and S4). The prodrugs were expressed in ExpiCHO cells, purified using protein A affinity chromatography and analyzed by SDS-PAGE and size exclusion chromatography, indicating no apparent aggregation (Figs S5 and S6). Thermal stability of the masked antibody construct was assessed using nano differential scanning fluorimetry (nanoDSF) and compared to nivolumab analyzed under identical conditions. The unfolding profiles showed similar melting transitions, with no apparent shift in melting temperature, indicating that fusion of the masking affibody domain does not adversely affect the thermal stability of the antibody (Fig. S7). The masking ability of the intact prodrugs compared to nivolumab was investigated using SPR by evaluating the reduction in binding to PD-1. Analysis of the ZnivoB9, ZnivoB5, and ZnivoA12-masked prodrugs revealed reduced PD-1 binding for all three constructs. Among the tested variants, ZnivoA12 exhibited slightly stronger PD-1 masking, while ZnivoB9 and ZnivoB5 displayed similar masking efficiency (Fig. 6b). The proteolytic activation of the prodrugs was evaluated using biolayer interferometry by analyzing their binding capacity to PD-1. Each prodrug was tested in both intact and cleaved forms to evaluate the effects of the masking domain and protease treatment on PD-1 binding (Fig. 6). Analysis of the masked prodrugs demonstrated a reduced PD-1 binding for all three constructs. Among them, ZnivoA12 displayed the strongest PD-1 masking, consistent with the results from the SPR analysis mentioned above. (Fig. 6c–e). Following protease cleavage, all three prodrugs regained PD-1 binding to levels comparable to the unmodified nivolumab control. Overall, these findings demonstrate that affibody-masked nivolumab prodrugs can effectively reduce PD-1 binding, and that protease cleavage restores their functional activity.

## Discussion

In this study, we aimed to develop affibody-based masking domains for nivolumab, an anti-PD-1 antibody used in cancer immunotherapy as a checkpoint inhibitor. The prodrug approach is motivated by the need to mitigate systemic immune activation and related toxicities, which are reported adverse effects of checkpoint blockade. By masking the complementarity-determining regions (CDRs) of nivolumab, we aimed to create a prodrug format with the potential for selective activation in the tumor microenvironment *via* specific proteases. While this study focused on the identification and *in vitro* validation of effective masking affibodies, our long-term goal is to engineer prodrugs that are activated specifically in tumors, thereby enhancing safety and therapeutic precision in clinical applications. Previous studies have demonstrated that affibody molecules can serve as effective masking domains in affibody-based prodrugs, where both the targeting and masking units are affibody molecules (Sandersjöö et al., 2015; Westerberg et al., 2025; Mestre Borras et al., 2023; Dahlsson Leitao et al., 2023). Here, we sought to explore whether affibody molecules could also function as masking domains for full-length monoclonal antibodies.

To this end, we screened an *E. coli*–displayed affibody library and identified several candidates capable of masking the CDRs of nivolumab, thereby preventing its interaction with PD-1. An unexpected and intriguing finding was the enrichment of proline-rich affibodies among the selected variants. Since proline residues are known to disrupt alpha helices, they were intentionally excluded from the original library design. The enrichment of these rare, proline-containing binders highlights the ability of the selection process to uncover unexpected yet functionally effective variants, including those that fall outside the intended sequence space. We hypothesized that the presence of prolines might disrupt one or more alpha helices within the affibody scaffold, potentially giving rise to a non-canonical structure that mimics PD-1. In a previously reported structure of the PD-1–nivolumab Fab complex, Tan and colleagues demonstrated that most of the interactions with nivolumab are mediated by a disordered N-loop in PD-1, which notably includes two proline residues (Tan et al., 2017). In our study, structural modeling using AlphaFold indicated a reduced alpha-helical content in the proline-rich affibodies. Moreover, the models provided additional insight into the mimicry mechanism, suggesting that these proline-enriched affibodies form interactions within the paratope of nivolumab, overlapping with the PD-1 binding site (Fig. 5). Flow cytometry and biolayer interferometry assays confirmed the functional capacity of these affibodies to block nivolumab’s PD-1 binding. In the prodrug setup, proteolytic cleavage restored nivolumab's interaction with PD-1, demonstrating the feasibility of using these affibody variants in a controlled activation mechanism.

The discovery of proline-rich affibodies as masking agents opens several intriguing avenues for future research. Their apparent structural mimicry of PD-1 highlights the potential to design epitope-specific anti-idiotypic masking domains that selectively target the CDRs of therapeutic antibodies. Future studies should investigate the stability and functionality of these affibodies *in vivo*, where the complexity of the tumor microenvironment and protease activity may differ substantially from *in vitro* conditions. In conclusion, our findings demonstrate that affibodies can be engineered to mask therapeutic antibodies such as nivolumab, offering a promising strategy to reduce systemic toxicity. The unexpected emergence of proline-rich affibodies and their mimicry of the PD-1 epitope provide new insights into affibody design. These results lay the groundwork for further exploration of target-mimicking peptides as masking domains, with the potential to enable safer and more effective antibody prodrugs.

## Supplementary Material

gzag010_Revised_Supplementary_material

## Data Availability

The data underlying this article are available in the article and in its online supplementary material.

## References

[ref1] Abramson J, Adler J, Dunger J et al. Accurate structure prediction of biomolecular interactions with AlphaFold 3. Nature 2024;630:493–500. 10.1038/s41586-024-07487-w. 38718835 PMC11168924

[ref2] Andersson KG, Persson J, Ståhl S et al. Autotransporter-mediated display of a naïve Affibody library on the outer membrane of Escherichia coli. Biotechnol J 2019;14:e1800359. 10.1002/biot.201800359. 30179307

[ref3] Berman HM, Westbrook J, Feng Z et al. The protein data Bank. Nucleic Acids Res 2000;28:235–42. 10.1093/nar/28.1.235. 10592235 PMC102472

[ref4] Dahlsson Leitao C, Mestre Borras A, Xu T et al. Conditionally activated affibody-based prodrug targeting EGFR demonstrates improved tumour selectivity. J Control Release 2023;357:185–95. 10.1016/j.jconrel.2023.03.046. 36990160

[ref5] Ferris RL, Blumenschein GJr, Fayette J et al. Nivolumab for recurrent squamous-cell carcinoma of the head and neck. N Engl J Med 2016;375:1856–67. 10.1056/NEJMoa1602252. 27718784 PMC5564292

[ref6] Guo L, Zhang H, Chen B. Nivolumab as programmed Death-1 (PD-1) inhibitor for targeted immunotherapy in tumor. J Cancer 2017;8:410–6. 10.7150/jca.17144. 28261342 PMC5332892

[ref7] Kavanaugh WM . Antibody prodrugs for cancer. Expert Opin Biol Ther 2020;20:163–71. 10.1080/14712598.2020.1699053. 31779489

[ref8] Löfblom J, Feldwisch J, Tolmachev V et al. Affibody molecules: Engineered proteins for therapeutic, diagnostic and biotechnological applications. FEBS Lett 2010;584:2670–80. 10.1016/j.febslet.2010.04.014. 20388508

[ref9] Lucchi R, Bentanachs J, Oller-Salvia B. The masking game: Design of Activatable Antibodies and Mimetics for selective therapeutics and cell control. ACS Cent Sci 2021;7:724–38. 10.1021/acscentsci.0c01448. 34079893 PMC8161478

[ref10] Meng EC, Goddard TD, Pettersen EF et al. UCSF ChimeraX: Tools for structure building and analysis. Protein Sci 2023;32:e4792. 10.1002/pro.4792. 37774136 PMC10588335

[ref11] Mestre Borras A, Dahlsson Leitao C, Ståhl S et al. Generation of an anti-idiotypic affibody-based masking domain for conditional activation of EGFR-targeting. New Biotechnol 2023;73:9–18. 10.1016/j.nbt.2022.12.002. 36526248

[ref12] Nilvebrant J, Hober S. The albumin-binding domain as a scaffold for protein engineering. in *computational and structural*. Biotechnol J 2013;6:e201303009. 10.5936/csbj.201303009. PMC3962080 24688717

[ref13] Parks L, Ek M, Ståhl S et al. Investigation of an AIDA-I based expression system for display of various affinity proteins on Escherichia coli. Biochem Biophys Res Commun 2024;696:149534. 10.1016/j.bbrc.2024.149534. 38241810

[ref14] Poreba M . Protease-activated prodrugs: Strategies, challenges, and future directions. FEBS J 2020;287:1936–69. 10.1111/febs.15227. 31991521

[ref15] Sandersjöö L, Jonsson A, Löfblom J. A new prodrug form of Affibody molecules (pro-Affibody) is selectively activated by cancer-associated proteases. Cell Mol Life Sci 2015;72:1405–15. 10.1007/s00018-014-1751-8. 25287047 PMC11113168

[ref16] Shimabukuro-Vornhagen A, Gödel P, Subklewe M et al. Cytokine release syndrome. J Immuno Therapy Cancer 2018;6:56. 10.1186/s40425-018-0343-9. PMC6003181 29907163

[ref17] Ståhl S, Gräslund T, Eriksson Karlström A et al. Affibody molecules in biotechnological and medical applications. Trends Biotechnol 2017;35:691–712. 10.1016/j.tibtech.2017.04.007. 28514998

[ref18] Tan S, Zhang H, Chai Y et al. An unexpected N-terminal loop in PD-1 dominates binding by nivolumab. Nat Commun 2017;6;8:14369. 10.1038/ncomms14369. PMC5303876 28165004

[ref19] Westerberg C, Mestre Borras A, Ståhl S et al. Affibody-based HER2 prodrug shows conditional cytotoxic effect on HER2-positive cancer cells. Biochem Biophys Res Commun 2025;758:151660. 10.1016/j.bbrc.2025.151660. 40117970

