# Supplementary material for: Engineering affibody domains as anti-idiotypic masks for nivolumab-based prodrugs
Source: Protein Eng Des Sel. 2026 Apr 16;39:gzag010. doi: 10.1093/protein/gzag010 (PMC13122598; doi:10.1093/protein/gzag010)

SUPPLEMENTARY MATERIAL

Figure S1. Representative variants from the high throughput analysis of nivolumab masking domains by flow cytometry. Flow cytometry analysis of representative affibody variants evaluated for nivolumab masking capacity. Affibody variants containing none, two, or three proline residues at randomized positions were selected from the high-throughput screening and tested for their ability to bind and mask nivolumab. Z_{wt} (binding to Fc) was included as a positive control for non-masked nivolumab binding. The upper panel shows nivolumab binding quantified by the median fluorescence intensity (MFI), while the lower panel shows the calculated masking capacity quantified by comparing binding to free nivolumab versus nivolumab pre-complexed with PD-1.

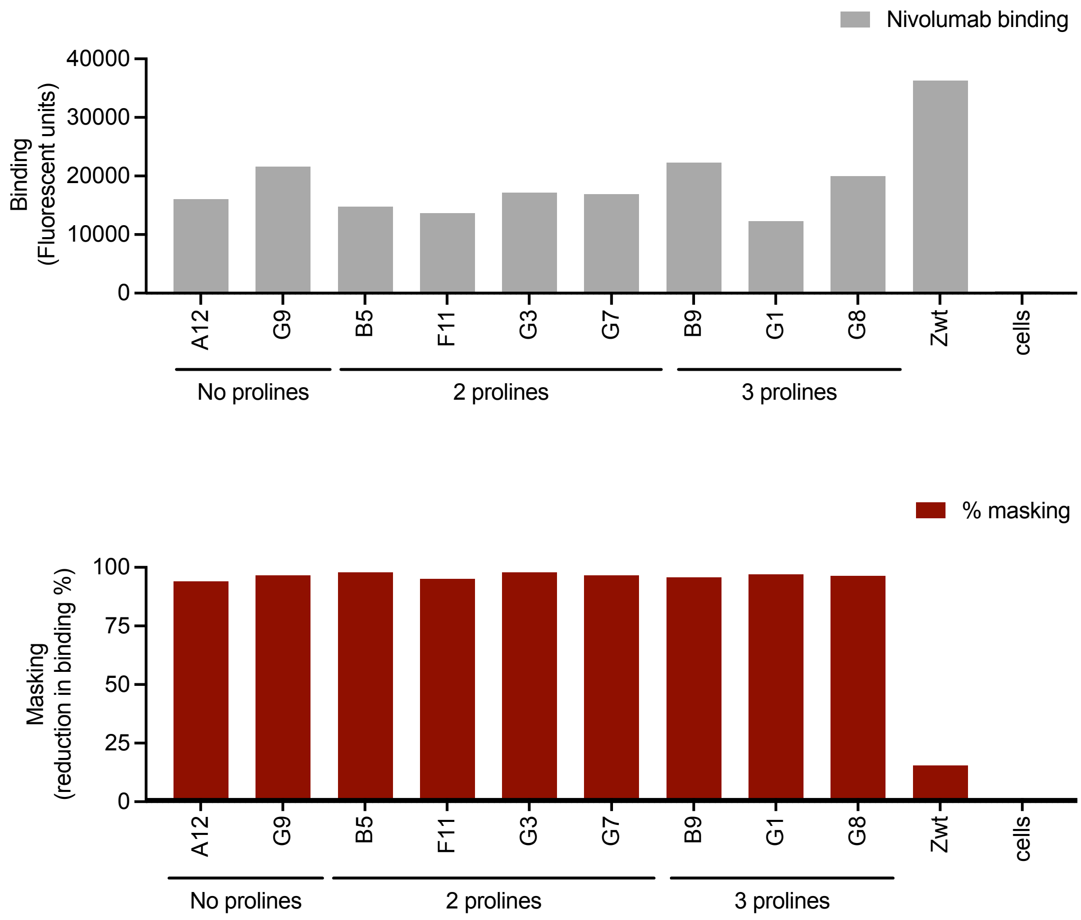

Figure S2. Sequences of selected candidates

	1	6	11	16	21	26	31	36	41	46	51	56
**ZnivoB5**	VDNKF	AKEQK	RAHRE	IHDLP	NLN**P**D	R**P**---	IRSLL	DDPSQ	SANLL	AEAKK	LNDAQ	APK
**ZnivoB9**	VDNKF	NKEHW	LAVHE	IFHPA	E**P**ERN	**P**DH**P**F	KSSLR	DDPSQ	SANLL	AEAKK	LNDAQ	APK
**ZnivoA12**	VDNKF	TKEKY	HAYAE	IRDLP	NLNQY	QKHAF	IASLH	DDPSQ	SANLL	AEAKK	LNDAQ	APK

Figure S3. Mass spectrometry analysis of purified soluble masking affibody candidates. Mass spectrometry of ZnivoB9, ZnivoB5, and ZnivoA12 confirmed the expected molecular weights

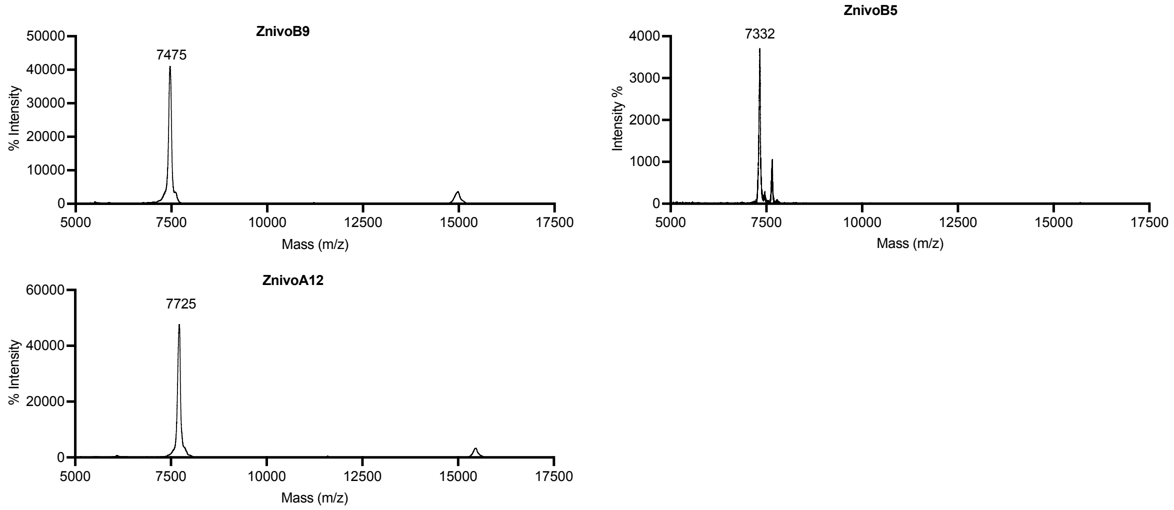

Supplementary Table 1. Nivolumab contact residues for PD-1 and affibody motifs

Interaction partner	Motif	Nivolumab residues (≤4 Å)*	Source
PD-1	P28–D29	N31, S32, G33, V50, W52, Y53, N99, D100	Crystal structure (PDB: 5WT9)
ZnivoB5	P24–D25	H35, V50, W52, N99	AlphaFold 3 model
ZnivoB9	P22–E23	G33, H35, V50, W52, Y53, N99	AlphaFold 3 model

*Residues within 4 Å of the indicated motifs are listed. PD-1 contacts are derived from a crystal structure (PDB: 5WT9), whereas ZnivoB5 and ZnivoB9 contacts are based on AlphaFold 3–predicted models.

Figure S4. Amino acid sequence of the nivolumab prodrug. The masking affibody is represented by candidate ZnivoB5 (green), which is fused to the N-terminus of nivolumab heavy chain (blue) by a linker (black). The TEV substrate is represented in red. The sequence shown includes the affibody, linker, TEV cleavage site, and the beginning of the nivolumab heavy chain variable domain.

V D N K F A K E Q K R A H R E I H D L P N L N P D R P I R S L L D D P S Q S A N L L A E A K K L N D A Q A P K G G G G S G G G G S E N L Y F Q G G G G S G G G G S Q V Q L V E S G G G V V Q P...

Figure S5. SDS-PAGE analysis of ZnivoB5–nivolumab prodrug before and after protease cleavage. Lane 1: molecular weight marker; Lane 2: parental nivolumab; Lane 3: parental nivolumab incubated with TEV protease (26 kDa); Lane 4: intact ZnivoB5–nivolumab prodrug, showing bands corresponding to the light chain (LC) and heavy chain fused to the affibody masking domain (Z HC); Lane 5: ZnivoB5–nivolumab prodrug treated with TEV protease, showing bands for cleaved heavy chain (HC), LC, and the released affibody masking domain (Z). Successful cleavage is indicated by the loss of the Z HC band and appearance of separate HC and Z bands.

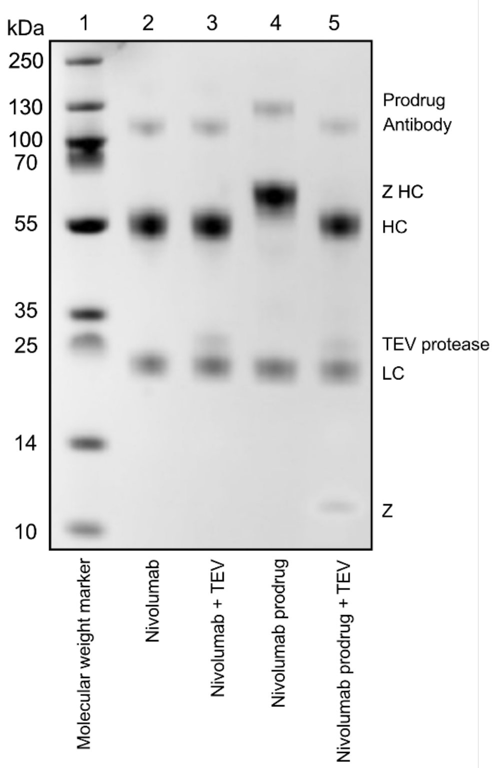

Figure S6. Size exclusion chromatography analysis of the prodrugs.
SEC chromatograms of nivolumab and affibody-masked prodrug constructs recorded on a Superdex 200 Increase column. UV absorbance at 280 nm is plotted as a function of elution volume.

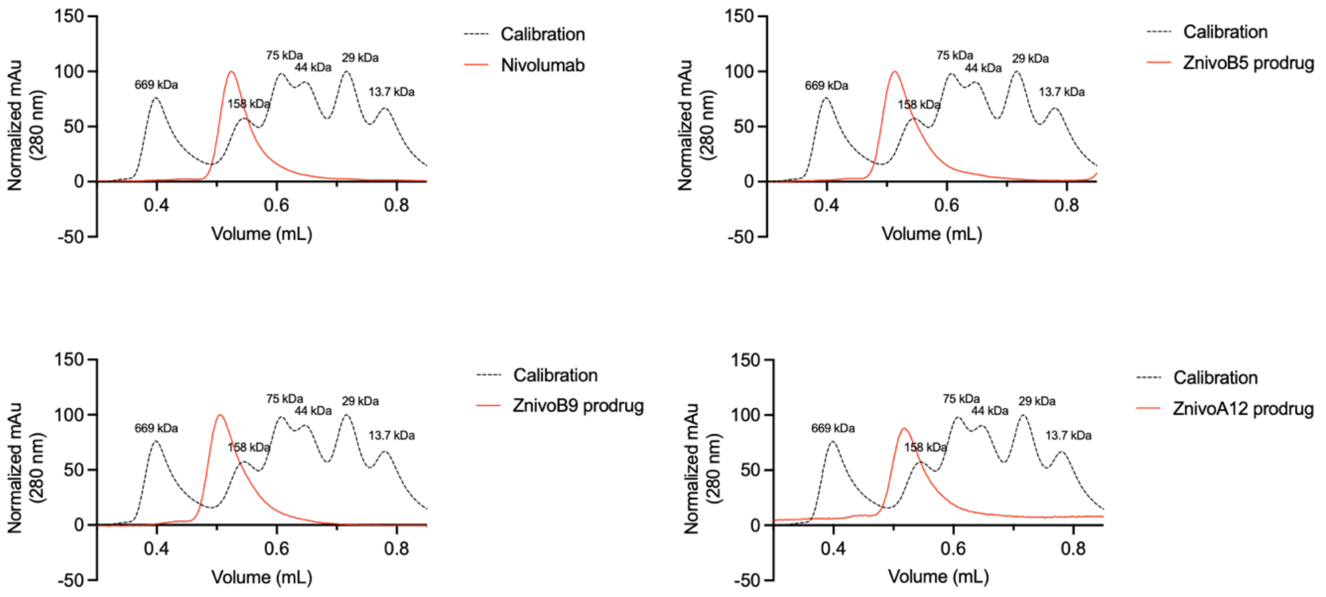

Figure S7. nanoDSF analysis of antibody constructs.
Unfolding transitions are shown as the first derivative of the intrinsic fluorescence ratio (F350/F330) with respect to temperature (dF/dT). Representative curves for the indicated constructs (Nivolumab control and ZnivoB9 prodrug) are shown, and the melting temperature (T_m) is indicated.

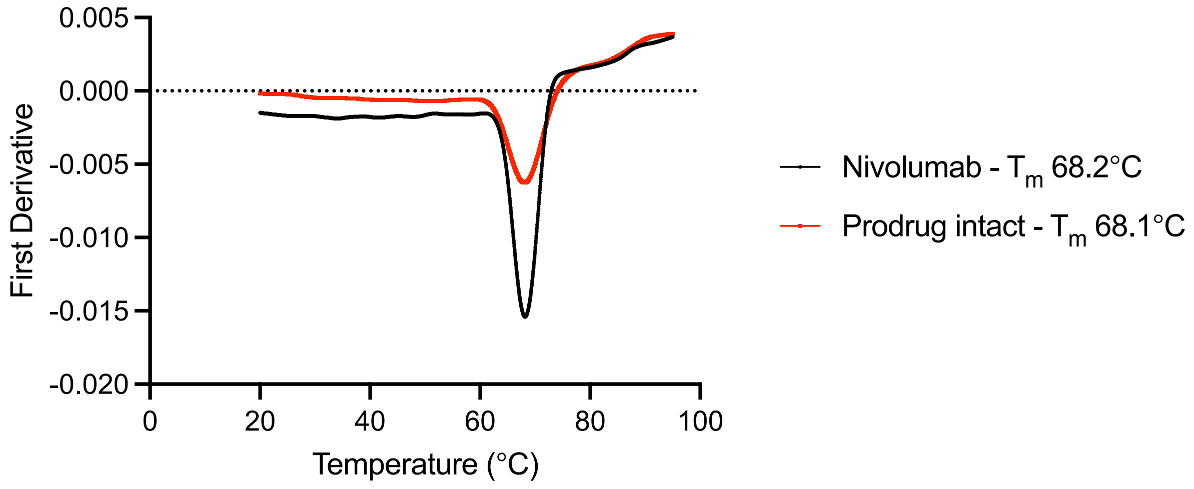

Supplement: gzag010_Revised_Supplementary_material [file gzag010_revised_supplementary_material.pdf]
